# Sperm SPACA6 protein is required for mammalian Sperm-Egg Adhesion/Fusion

**DOI:** 10.1038/s41598-020-62091-y

**Published:** 2020-03-24

**Authors:** Sandrine Barbaux, Côme Ialy-Radio, Myriam Chalbi, Elisa Dybal, Méline Homps-Legrand, Marcio Do Cruzeiro, Daniel Vaiman, Jean-Philippe Wolf, Ahmed Ziyyat

**Affiliations:** 1 Université de Paris, Institut Cochin, INSERM, CNRS, F-75014 PARIS, France; 2 Service d’Histologie, d’Embryologie, Biologie de la Reproduction, AP-HP, Hôpital Cochin, F-75014 PARIS, France

**Keywords:** Developmental biology, Medical research

## Abstract

Three genes are known to be essential for gamete adhesion/fusion (*Cd9, Izumo1* and *Juno*). Here, we confirmed that *Spaca6* null males are infertile and showed that their sperm accumulate in the perivitelline space but are unable to fuse with oocyte. Like IZUMO1, SPACA6 which is expressed by human sperm, is remained on the equatorial segment after acrosomal reaction and is involved in human fertilization since an anti-SPACA6 antibody inhibited it. Despite the similarity of the phenotypes caused by *Spaca6* and *Izumo1* knockouts, these are not redundant and the essential relocation of IZUMO1 is not affected by the lack of SPACA6. We propose a model in which IZUMO1 and SPACA6 would be part of a molecular complex necessary for gamete fusion and that their concomitant presence would be required for the recruitment of another essential molecular actor, such as a fusogen, for the fusion to take place.

## Introduction

Among the membrane molecular players involved in mammalian gamete adhesion/fusion, only three, the spermatic immunoglobulin IZUMO1^1^, the glycosylphosphatidylinositol-anchored protein (GPI-AP) JUNO, its oocyte receptor^2^ and the oocyte CD9 tetraspanin are described as essential^3–5^. These three proteins are also present on human gametes and play an important role during fertilization^1,6–8^.

CD9 orchestrates the adhesion molecular complex on the oocyte side. A biophysical approach, a strategy consisting in a direct measurement of the force necessary to separate one sperm in contact with an egg, demonstrated that CD9 induces a pre-fusional adhesion suggesting that the fusion defect in the case of *Cd9* deletion may originate from an altered adhesion and not from an altered fusion^9^. The fact that CD9 contains no fusogenic peptide reinforces this idea. The surface of the mouse oocyte can be divided into two regions: a microvillar large region and an amicrovillar small region. The binding and fusion of sperm take place mainly in the oocyte region rich in microvilli, emphasizing its importance for sperm-oocyte fusion^10,11^. Furthermore, the mouse oocyte microvillar region is enriched in CD9^10^. Moreover, the contact zone during fertilization is enriched concomitantly with CD9, JUNO and IZUMO1^8,12,13^. The cylindrical shape and narrow diameter of the microvilli provide the small radius of curvature necessary to overcome the repulsive interactions between cell surfaces during fusion. While a small radius of curvature promotes fusibility, that of the microvillar tips of *Cd9* null oocytes has been found to be twice as large as that of wild-type oocytes^10^.

IZUMO1 N-terminal region is important for its binding to the egg membrane^2,6^, however, and according to Inoue *et al*., IZUMO1 is monomeric in sperm and it is the adhesion of sperm with an oocyte that triggers its dimerization^12^. Using COS-7 cells expressing IZUMO1, Inoue *et al*. observed their binding to eggs and the accumulation of IZUMO1 at the interface between the two cells, but without fusion^6^. Therefore, it is likely that IZUMO1 may interact with other proteins on the sperm membrane which could facilitate the fusion process itself^14^, as suggested by co-immunoprecipitation studies^15^. Using cells that did not spontaneously adhere to the egg plasma membrane (namely K562) transfected to express IZUMO1, it was shown that they developed an adhesion with the eggs and that the forces linking the membranes together were strong enough to resist tractions, however without fusing^16^. More recently, JUNO exclusion at the surface of COS-7 cells expressing JUNO was observed when in contact with cells expressing IZUMO1, suggesting the existence of an unidentified oocyte receptor, which may be a general cell adhesion molecule such as an integrin. However, the possibility that this factor could be a non-protein factor such as a phospholipid was not excluded^12^. JUNO exclusion of the contact area may be similar to CD9 and α6 integrin exclusion observed at the time of adhesion/fusion^8^. Ravaux *et al*. have reported that these depletions take place after the fusion stage^13^. This result was made possible by the use of a microfluidics approach which, combined with relevant fluorescent probe, guarantiees the best frontal view of the sperm/egg interaction area and the timeline of the fertilization events. Thanks to this approach, these authors have also added a new element in the understanding of the stages of gamete adhesion/fusion. Indeed, they revealed that the oscillatory movement of the sperm head on the oocyte plasma membrane, due to a specific flagellum-beating mode, is a crucial element of adhesion phase to initiate fusion^17^ and that these specific sperm oscillations also induce CD9 concentration at the egg/sperm interface until a CD9-rich platform is nucleated on which fusion immediately takes place^13^.

Regarding the protein elements, there are certainly other actors to discover since, after the invalidation of one of the three known essential genes in mice, there is virtually no fusion, but adhesion persists, suggesting the existence of other molecular players involved in adhesion stage. And in particular on the sperm side where only one protein (IZUMO1) has been described as essential. In this context, the random integration of a transgene, that has been reported to result in a deletion on mouse chromosome 17, encompasses two genes (*Has1* and *Spaca6*) but inactivates only an immunoglobulin superfamily gene (*Spaca6*), potentially involved in sperm–egg adhesion/fusion. Indeed, male mice harboring this deletion were completely sterile^18^. The observed phenotype was similar to that caused by the deletion of *Izumo1*, *Cd9* or *Juno*, namely infertility and the accumulation of sperm in the perivitelline space (PVS). Human *SPACA6* on chromosome 19, also named *BACHELOR-like*, was originally thought to be a non-coding gene. Now it is known that this gene encodes an immunoglobulin-like protein, a single-pass type I membrane protein containing a signal peptide (aa 1 to 26), a large extracellular domain (aa 27 to 295), a transmembrane helical domain (aa 296 to 316) and small cytoplasmic domain (aa 317 to 324).

Here, we confirmed the testis expression of SPACA6 in mouse and human and the presence of SPACA6 on human sperm where, like IZUMO1, it relocalizes during the sperm acrosome reaction. A polyclonal anti-hSPACA6 antibody was capable of inhibiting human IVF. Then we conducted a CRISPR/Cas9 mutagenesis and generated two different genetic background mouse lines invalidated for *Spaca6* that have the same phenotype, namely the total sterility of males due to sperm inability to fuse with oocyte and their accumulation in PVS *in vivo* and *in vitro*. This fertilizing defect of *Spaca6* deleted sperm was rescued by ICSI. Finally, we demonstrated that the absence of *Spaca6* has no effect on *Izumo1* sperm localization and its essential relocation after physiological acrosome reaction. In short, there is no longer any doubt about the essential nature of SPACA6’s role in fertilization.

## Results

### Human SPACA6 is detectable on sperm head after acrosome reaction

In order to localize SPACA6 in mouse and human sperm, antibodies directed against the mouse and human forms of SPACA6 proteins were produced in rabbits. The antibody directed against the mouse protein was completely non-specific since the same signals were found both in Western Blot and in immunofluorescence on *Spaca6* transfected and non-transfected cells. This negative result was confirmed when we used WT and *Spaca6* deleted sperm (data not shown).

**Figure 1:**
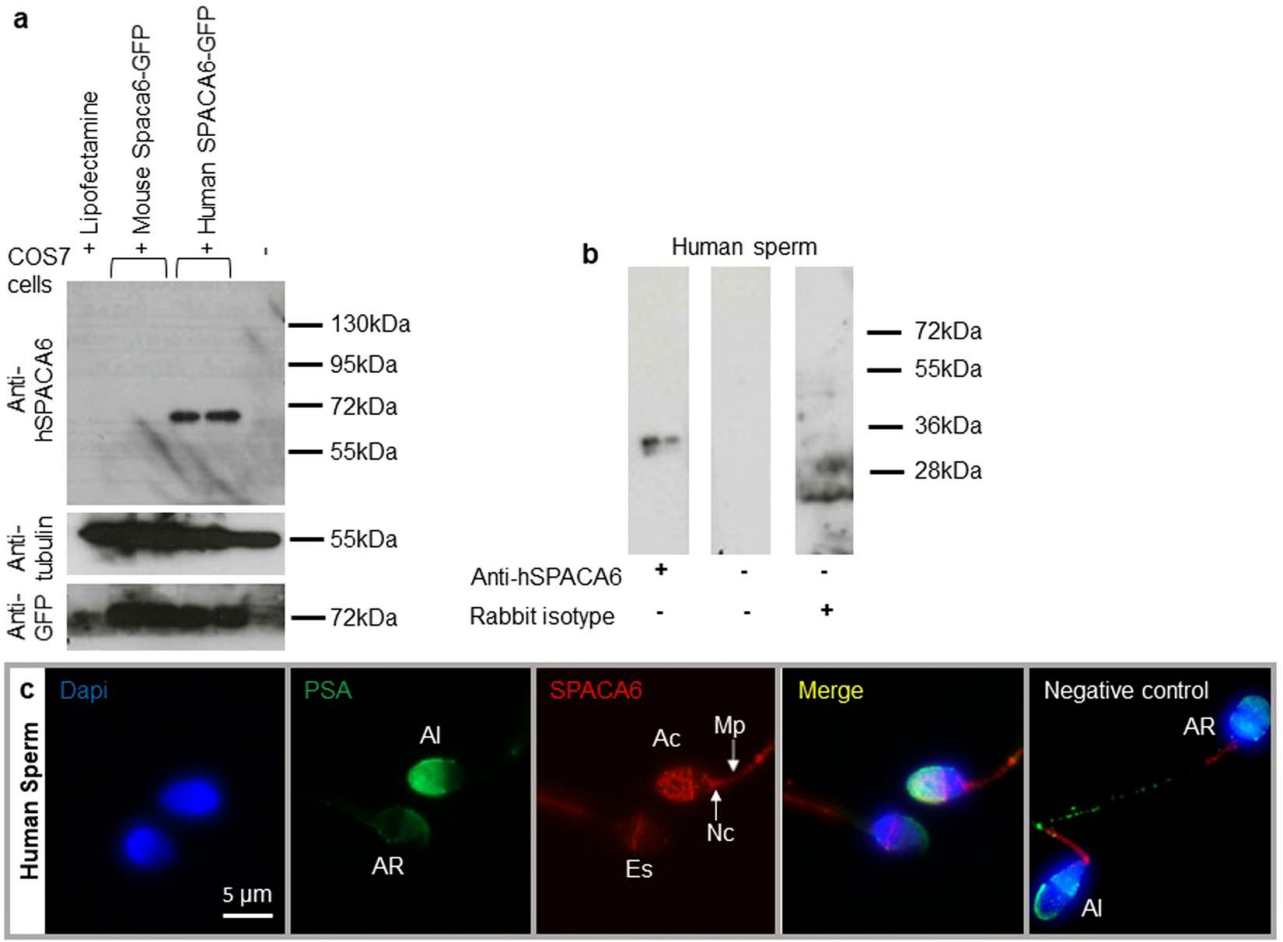
Human sperm SPACA6 expression. (**a)** Western blot using extracts of COS-7 cells transfected or not with mouse *Spaca6*-GFP and human *SPACA6*-GFP, revealed with rabbit polyclonal anti-human SPACA6, anti-Tubulin and anti-GFP antibodies. A band about 72 kDa (~36 kDa for SPACA6 + ~36 kDa for GFP) was visible only in COS-7 human *SPACA6*-GFP samples. In contrast, all samples were positive for Tubulin (55 kDa band) and those transfected with GFP were positive for it, demonstrating the quality of the deposited proteins and the efficiency of transfection respectively.(**b)** Western blot of human sperm protein extract using the anti-hSPACA6 antibody. The expected size band (~36 kDa) is only in the antibody lane. Controls with secondary antibody alone or with rabbit isotype (IgG) were negative. The corresponding full-length blots are presented in Supplementary Fig. S1. (**c**) Human SPACA6 distribution on sperm. Rabbit polyclonal anti-hSPACA6 revealed by a Donkey anti-rabbit Alexa 594 conjugated secondary antibody (red), localized SPACA6 on the acrosomal cap, equatorial and neck regions and midpiece of acrosomal intact sperm as attested by positive PSA-FITC conjugated (green) staining. After acrosome reaction (PSA-FITC negative), SPACA6 was remained essentially in the equatorial segment of sperm head. Nucleus was stained with Dapi (blue). AI: Acrosome Intact, AR: Acrosome Reacted, Es: Equatorial segment, Ac: Acrosomal cap, Nc: Neck, Mp: Midpiece.

Only the antibody designed against the human protein proved to be specific using transfected COS-7 Cells. As expected, this antibody was specific of the human form of SPACA6. Indeed, it does not recognize the mouse form of the protein even if its expression was revealed by an anti-GFP antibody (Fig. 1a). This antibody was then used for Western blot, immunofluorescence and *in vitro* fertilization experiments in human. Figure 1b illustrates by Western blot analysis the presence of an expected ~36 kDa band corresponding to human SPACA6 protein size on sperm samples. SPACA6 was not detected by immunofluorescence in fresh sperm with intact acrosome (data not shown). After permeabilization treatment or at least fixation which induces a light permeabilization, observation allowed us to localize SPACA6 on the membrane of sperm head at the acrosomal cap in acrosome intact sperm as attested by colocalization with FITC conjugated-Pisum sativum Agglutinin lectin (PSA) that exclusively stains the intact acrosome (Fig. 1c). SPACA6 is also localized at equatorial region, neck, and midpiece. Interestingly, after acrosomal reaction (negative for PSA staining), SPACA6 remained essentially at the level of the equatorial segment, became weak at the level of the midpiece and disappeared from the acrosomal cap and the neck region (Fig. 1c). Negative control showed a residual staining at midpiece in intact and reacted acrosome sperm indicating that this staining is likely nonspecific. As IZUMO1^1^, SPACA6 seems to be an acrosomal membrane protein that is not exposed before the completion of an acrosome reaction.

### SPACA6 is involved in Human fertilization

**Figure 2:**
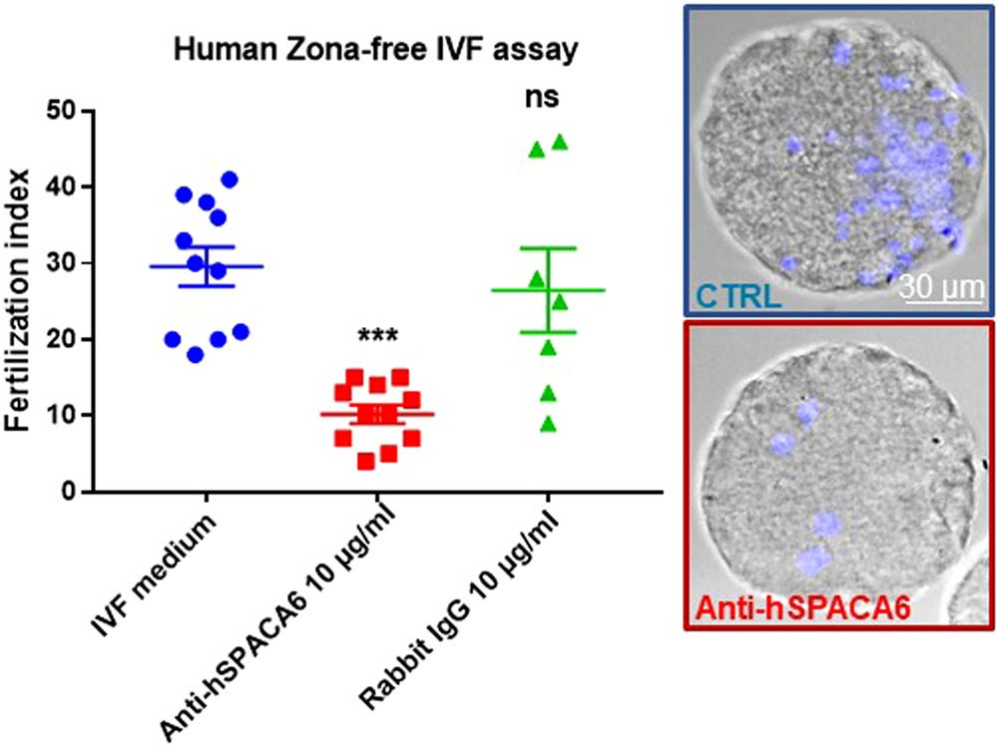
Anti-hSPACA6 antibody inhibited Human fertilization. Overnight (18 h) insemination of zona-free human oocytes with human sperm in the presence of rabbit polyclonal anti-hSPACA6 (10 µg/ml), or rabbit IgG (10 µg/ml) or only IVF medium. The number of fused sperm heads into egg cytoplasm was recorded after insemination under fluorescent microscope (Nikon eclipse E600) and the fertilization index (FI: Number of decondensed sperm per oocyte, mean ± SEM) was calculated. FI decreased drastically from 29.5 ± 2.5 (n = 11) for the control group to 10.1 ± 1.1 (n = 11) for antibody treated group (10 µg/ml). No significant difference was observed between IVF medium control and IgG (at 10 µg/ml) control groups (FI: 26.4 ± 5.5 (n = 7)). Examples of illustrative images obtained after confocal analysis (Spinning Disk) are shown for the control and antibody groups. Transmission images were superimposed with Hoechst fluorescent signals (blue).

To assess the involvement of SPACA6 in human fertilization, we used zona-free *in vitro* fertilization assay in presence of a rabbit polyclonal anti-SPACA6 antibody. We used this test because cumulus-intact IVF is prohibited in France by bioethics law. Removing the zona pellucida allows polyspermia and therefore we measured the fertilization index (FI), i.e. the number of sperm fused per oocyte. As shown in Fig. 2, the fertilization index decreased drastically from 29.5 ± 2.5 (n = 11) for the control group to 10.1 ± 1.1 (n = 11) sperm fused per oocyte for the antibody treated group (10 µg/ml). No significant difference was observed between control and IgG groups at 10 µg/ml (FI: 26.4 ± 5.5 (n = 7)). These results show that sperm SPACA6 is involved in gamete membrane adhesion/fusion during human fertilization.

### *Spaca6* gene deleted male mice were sterile

**Figure 3:**
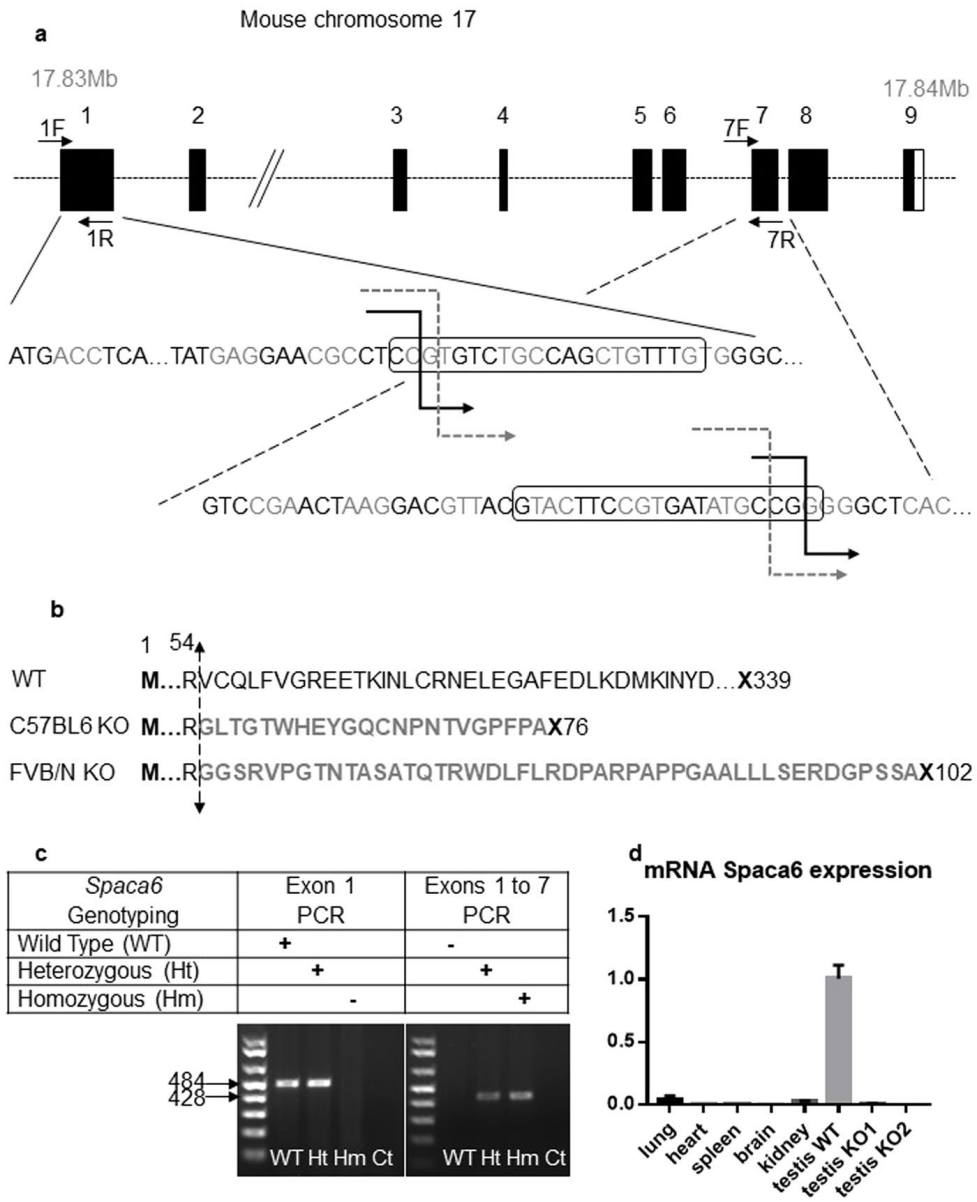
CRISPR-Cas9 *Spaca6* gene deletion. (**a**) Genic organization of the mouse *Spaca6* gene corresponding to cDNA NM_001162909. Black boxes are coding exons; the white box is the 3′ untranslated region. Black broken arrows: cuts in the C57BL/6 background. Grey dotted broken arrows: cuts in the FVB/N background. Sequences of the guides in exons 1 and 7 are in boxes. (**b**) Peptide sequences of the SPACA6 protein in the WT conditions (339 aa) and in both deleted versions: the deletion maintains the first 54 aa, then introduces 22 or 45 new different aa before a premature stop codon in the C57BL/6 and FVB/N background respectively. (**c**) Genotyping was performed by PCR on tail-tip DNA using *Spaca6* Exon 1 F and 1 R primers and *Spaca6* Exon1F and Exon 7 R. This last pair of primers gives a band (~428 bp) only when the deletion between exons 1 and 7 has taken place. (**d**) The absence of *Spaca6* expression was verified by RT-qPCR. No transcripts were detected in the testis of two KO males, unlike the WT testis. Several WT tissues (lung, heart, spleen, brain and kidney) have also been shown to be negative for *Spaca6* expression.

Located on mouse chromosome 17, *Spaca6* is a 18 kb gene containing 9 exons. To analyze the physiological role of *Spaca6*, we generated two mutant mice by the CRISPR/Cas9 procedure. Using two different CRISPR sites, one in exon 1 and one in exon 7, this strategy allowed to induce the deletion of a 7 kb fragment when both disruptions occur (Fig. 3a). C57BL/6J and FVB/N mice zygotes have been injected with both sgRNAs and Cas9 protein. Deletions from exon 1 to exon 7 were confirmed by PCR and direct sequencing analysis of PCR products (Fig. 3c). This large deletion caused a loss of most of the ORF and a premature termination codon (Fig. 3b). RT-qPCR analysis of *Spaca6* transcripts in *Spaca6* homozygous knockout (KO) mouse testis was negative unlike for WT testis. Several WT tissues (lung, heart, spleen, brain and kidney) have also been shown to be negative for *Spaca6* expression (Fig. 3d).

No deleterious effect on testicular histology was observed in KO males and sperm produced by *Spaca6* KO males were motile and morphologically normal under phase-contrast microscopy (data not shown).

**Figure 4:**
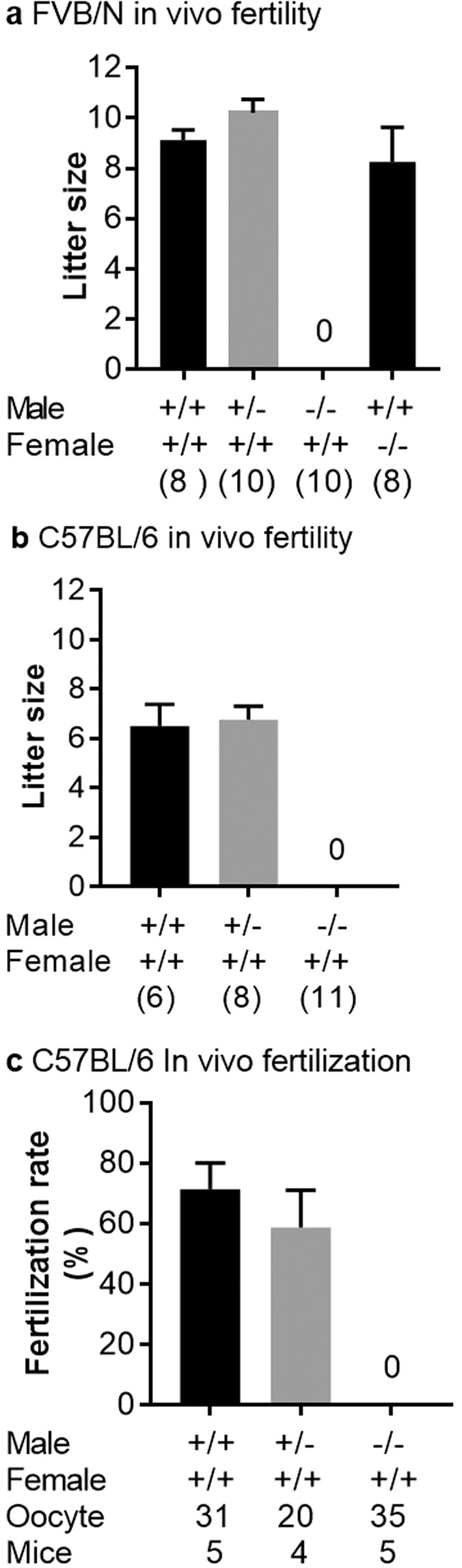
*In vivo* sterility of *Spaca6* KO males. Litter size of crosses between FVB/N (**a**) or C57BL/6 (**b**) females and WT, heterozygotes, and KO males. Values indicate mean ± SEM. For both genetic backgrounds, no difference between WT and heterozygous males was found and no birth was obtained with KO males. FVB/N KO females were normally fertile (**a**). The numbers in parentheses indicate the numbers of mating pairs. (**c**) The same results were obtained when the fertilization rate of oocytes recovered in the oviduct the day after mating (as attested by the presence of a vaginal plug) was measured.

To examine male fertility, KO adult males (FVB/N and C57BL/6) were mated with wild-type females. KO males were sterile, though showing normal mating behavior with vaginal plug formation. The mean litter size was 9.1 ± 0.3 with WT males, 10.2 ± 0.5 with heterozygous males and no pups were obtained with KO males while FVB/N KO females were normally fertile (8.2 ± 1.3) for the FVB/N line (Fig. 4a). For the C57BL/6J line, the mean litter size was 6.5 ± 0.88 with WT males, 6.7 ± 0.5 with heterozygous males and no pups were obtained with KO males (Fig. 4b). With less data than for FVB/N females, C57BL/6 KO females also appeared to be normally fertile (data not shown). These data confirmed that *Spaca6* is essential for male fertility in mice.

### *Spaca6* deleted mice sperm were unable to fertilize oocytes *in vivo*

Crosses with WT females showed that *Spaca6* KO males were sterile, it remained to specify the stage of fertilization impacted by this deletion. We then crossed *Spaca6* KO males with WT females and recovered the oocytes (or embryos) in order to check their status in terms of fertilization. While WT males gave a fertilization rate of 71.4 ± 8,6 and heterozygous males gave 58.8 ± 12.3, no fertilization was obtained with KO males (Fig. 4c). But in the latter case, spermatozoa were found swimming in the PVS (Video S1). This phenotype was found *in vivo* and *in vitro* in both KO lines (C57BL/6J and FVB/N). This result indicated above all that all the steps preceding the membrane adhesion/fusion step proceeded normally since the spermatozoa were able to migrate in the female genital tract until they reached the fertilization site, the oviduct. Then they were able to interact with and cross the two barriers that protect the oocyte, the cumulus cells and the zona pellucida. This experiment suggested the step of adhesion/fusion as being at the origin of the sterility of the *Spaca6* KO males.

### *In vitro* accumulation of sperm of *Spaca6* deleted mice in the perivitelline space of the eggs

**Figure 5:**
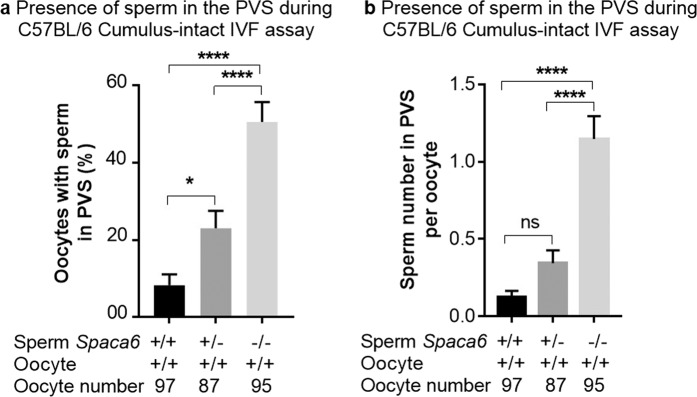
Abnormal frequency of *Spaca6* KO sperm presence in the perivitelline space. After cumulus-intact IVF using sperm from *Spaca6*+*/+, Spaca6*+*/− or Spaca6−/−* C57BL/6 males, were reported: (**a**) the percentage of perivitelline space sperm containing-oocytes (mean ± SEM) which showed high and significant difference when comparing the KO to heterozygotes or WT groups (P < 0.0001) and less but significant difference when comparing heterozygotes and WT groups (P < 0.05), or (**b**) sperm number in perivitelline space per oocyte (mean ± SEM). In this case, only the difference between KO sperm and the two other groups was significant (P < 0.0001).

This phenotype of sperm presence in PVS was then quantified *in vitro* after cumulus-intact IVF using sperm derived from the C57BL/6J KO males (Video S2) and compared with those from heterozygous and WT males. The presence of sperm in PVS exists also with WT sperm and oocytes. Indeed, the percentage of WT oocytes containing sperm in PVS from WT males was 8.2 ± 2.8% (n = 97) while this percentage increased to 22.9 ± 4.5% (n = 87, *P* = 0.27) with sperm from *Spaca6* heterozygous males and to 50.5 ± 5.1% (n = 95) with sperm from *Spaca6* KO males (*P* < 0,0001 when compared with both WT or heterozygous groups) (Fig. 5a).

When we considered the number of sperm per oocyte, we obtained a mean of 0.12 ± 0.04, 0.34 ± 0.08 and 1.14 ± 0.14 for WT, heterozygous and KO groups respectively (Fig. 5b). The statistic difference between KO group and the two others was very significant (*P* < 0.0001). Between the WT and the heterozygous groups, the difference was less important (*P* < 0,04).

### *Spaca6* KO sperm do not fertilize *in vitro*

**Figure 6:**
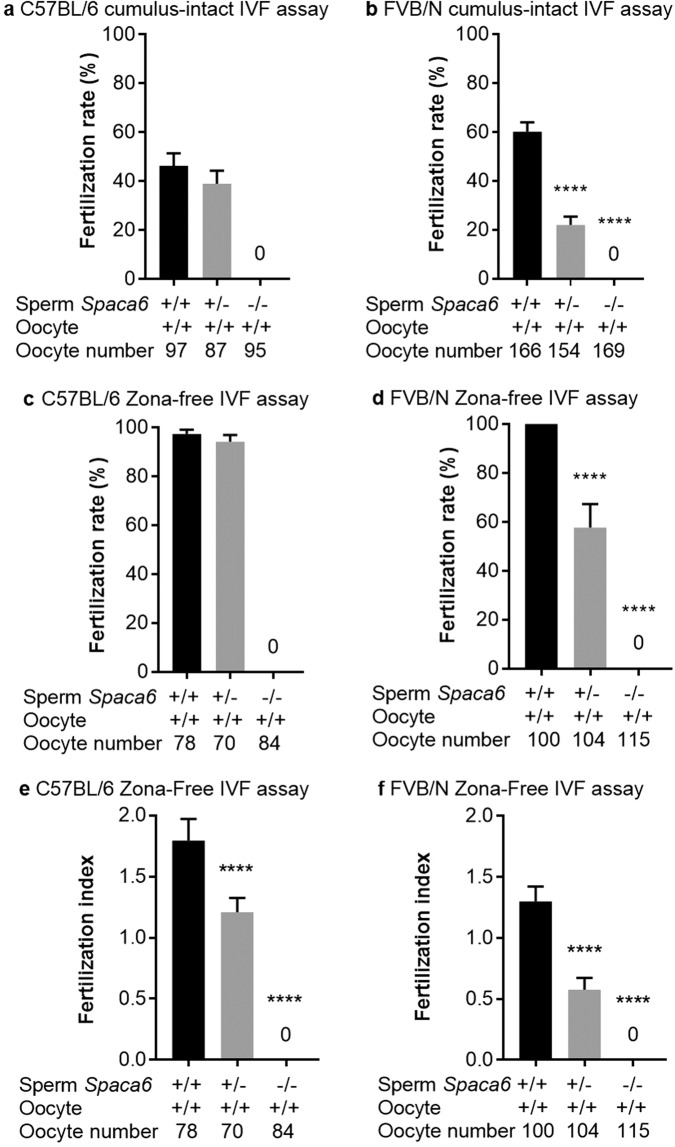
No fertilization was obtained with *Spaca6* KO sperm *in vitro*. Fertilization rate (FR) (mean ± SEM) following cumulus-intact IVF assay at 10^6^ sperm per ml or zona-free IVF assay at 10^5^ sperm per ml for 3 hours. No fertilization was obtained with *Spaca6* KO sperm in C57BL/6 (**a**) and FVB/N (**b**) lines. While no differences concerning the FR were observed when comparing the WT and heterozygous groups in C57BL/6 background (**a,c**), significant ones were obtained in FVB/N background both in cumulus-intact (**b**) and zona-free assays (**d**) (P < 0.0001). Regarding the fertilization index (FI; mean ± SEM), measurable only in zona-free IVF assay, and that gives the average number of fused sperm per oocyte observed after insemination, significant differences were observed in both backgrounds (**e** for C57BL/6 and **f** for FVB/N) when heterozygous groups were compared to WT ones (P < 0.0001). The sperm and oocyte *Spaca6* genotypes are indicated below each figure.

To investigate the ability of *Spaca6* KO sperm to fertilize *in vitro*, we performed cumulus-intact and zona-free *in vitro* fertilization (IVF) assays using the FVB/N and C57BL/6J background KO males and their respective heterozygous and WT controls. For cumulus-intact IVF assay with the C57BL/6J line, we obtained a fertilization rate (FR) of 46.3 ± 5, and 39 ± 5.2 (*P* = 0.3) with WT and heterozygous sperm respectively, while no fertilization was obtained with KO sperm (Fig. 6a and Video S3). On the FVB/N background, sperm from KO males still did not show any fertilization, but a significant difference was observed between WT sperm (FR: 60.2 ± 3.8) and heterozygous sperm (FR: 22 ± 3.3, *P* < 0,0001) (Fig. 6b). This difference in term of fertilization rate was confirmed in zona-free assays between FVB/N WT and heterozygous sperm (Fig. 6d) while no difference was obtained on C57BL/6J background (Fig. 6c). On the other hand, a difference between the heterozygous and the WT groups appeared on both genetic backgrounds when we compared the fertilization indexes (FI). Indeed, FI of 1.79 ± 0.08, 1.20 ± 0.05 and 0 were obtained for C57BL/6J WT, heterozygotes and KO respectively (P < 0,0001; Fig. 6e) and FI of 1.3 ± 0.06, 0.57 ± 0.04 and 0 were obtained for FVB/N WT, heterozygotes and KO respectively (P < 0,0001; Fig. 6f).

### ICSI rescues the fertilizing ability of *Spaca6* deleted sperm

No offspring was obtained by *Spaca6* deleted male mice and no fertilization was obtained *in vitro* with *Spaca6* deleted sperm and in the two cases we observed the presence of sperm in the PVS suggesting that SPACA6 is essential for the gamete adhesion/fusion step. To confirm this, we used intracytoplasmic sperm injection (ICSI) to insert *Spaca6* KO sperm directly into the cytoplasm of wild-type oocytes, bypassing the adhesion/fusion step. Oocytes injected with *Spaca6* deleted or with WT sperm were successfully fertilized and transplanted into the oviducts of pseudopregnant females. The eggs implanted and embryos developed normally with similar rates in the two groups (Table 1).

**Table 1:** ICSI rescues the fertilizing ability of *Spaca6* deleted sperm.

Male	Oocyte number	Survived eggs after ICSI	Eggs at 2-cell stage	Number of pups
*Spaca6* +/+	47	38	34 (89%)*	5 (15%)^†^
*Spaca6* −/−	48	43	25 (58%)*	3 (12%)^†^

*Percentages are based on numbers of eggs surviving after ICSI.

^†^Percentages are based on numbers of eggs at 2-cell stage.

### The absence of SPACA6 has no effect on IZUMO1 sperm localization and relocation after acrosome reaction

**Figure 7:**
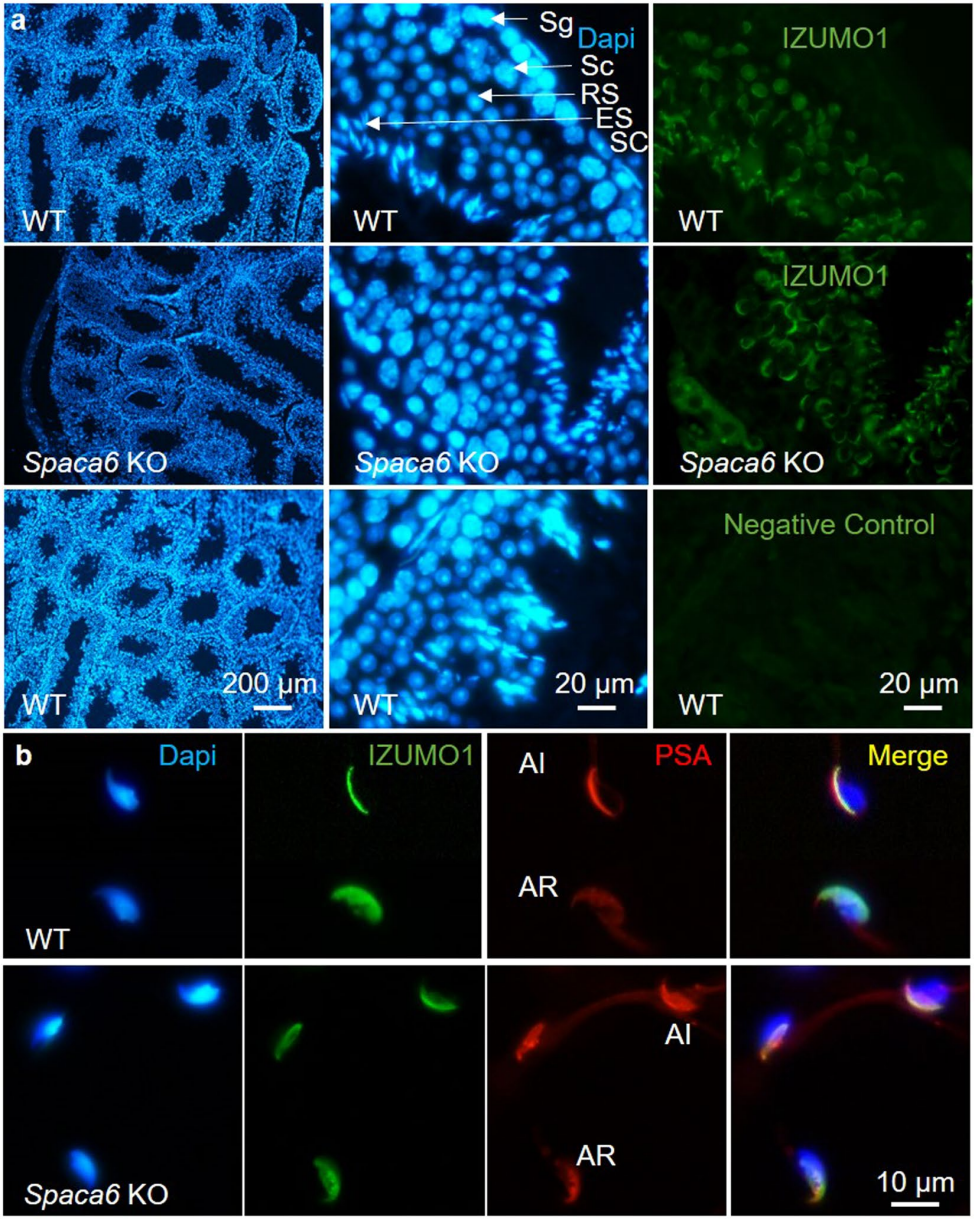
Normal testis and epididymal sperm localization and relocation after acrosomal reaction of IZUMO1 in *Spaca6* KO context. (**a**) Immunofluorescence staining of IZUMO1 and DAPI staining of permeabilized testicular sections from WT and *Spaca6* KO C57BL/6 mice. The labeling of IZUMO1 (green) was carried out using a rat monoclonal anti-mouse IZUMO1 primary antibody, recognized by a secondary anti-rat goat antibody coupled to Alexa Fluor 488. The nuclei of the testicular cells were labeled with DAPI at 10 μg/mL. IZUMO1 was similarly detected in round and elongated spermatids (RS and ES) from WT and *Spaca6* KO testes. A control condition with an incubation of the tissue with the secondary antibody alone was performed to check the specificity of the antibodies and to evaluate the background noise (negative control). Sg: Spermatogonia, Sc: Spermatocyte, RS, Round spermatid, ER: Elongated Spermatid, SC: Sertoli Cell. (**b**) Immunofluorescence staining of IZUMO1 and DAPI and PSA labeling of epididymal sperm from WT and *Spaca6* KO males before or after acrosome reaction. While IZUMO1 is essentially localized in acrosomal cap in acrosome-intact sperm, it is more distributed on the sperm head after acrosome reaction. The absence of SPACA6 has no effect on IZUMO1 sperm localization and relocation after acrosome reaction.

Since the phenotype of *Spaca6* KO was identical to that of *Izumo1* KO, it was legitimate to investigate the question of the link between these two proteins. We therefore investigated whether the absence of SPACA6 had an effect on the localization of IZUMO1. As illustrated in Fig. 7a, we observed no difference on the testis localization of IZUMO1 in WT and KO male mice. As expected, IZUMO1 is detected from the round spermatid stage, on the acrosome. Then we compared by direct immunofluorescence staining IZUMO1 localization on epididymal sperm of WT and *Spaca6* KO mice before and after sperm acrosome reaction and concluded that the absence of SPACA6 has no effect on IZUMO1 sperm localization and relocation after acrosome reaction (Fig. 7b).

**Figure 8:**
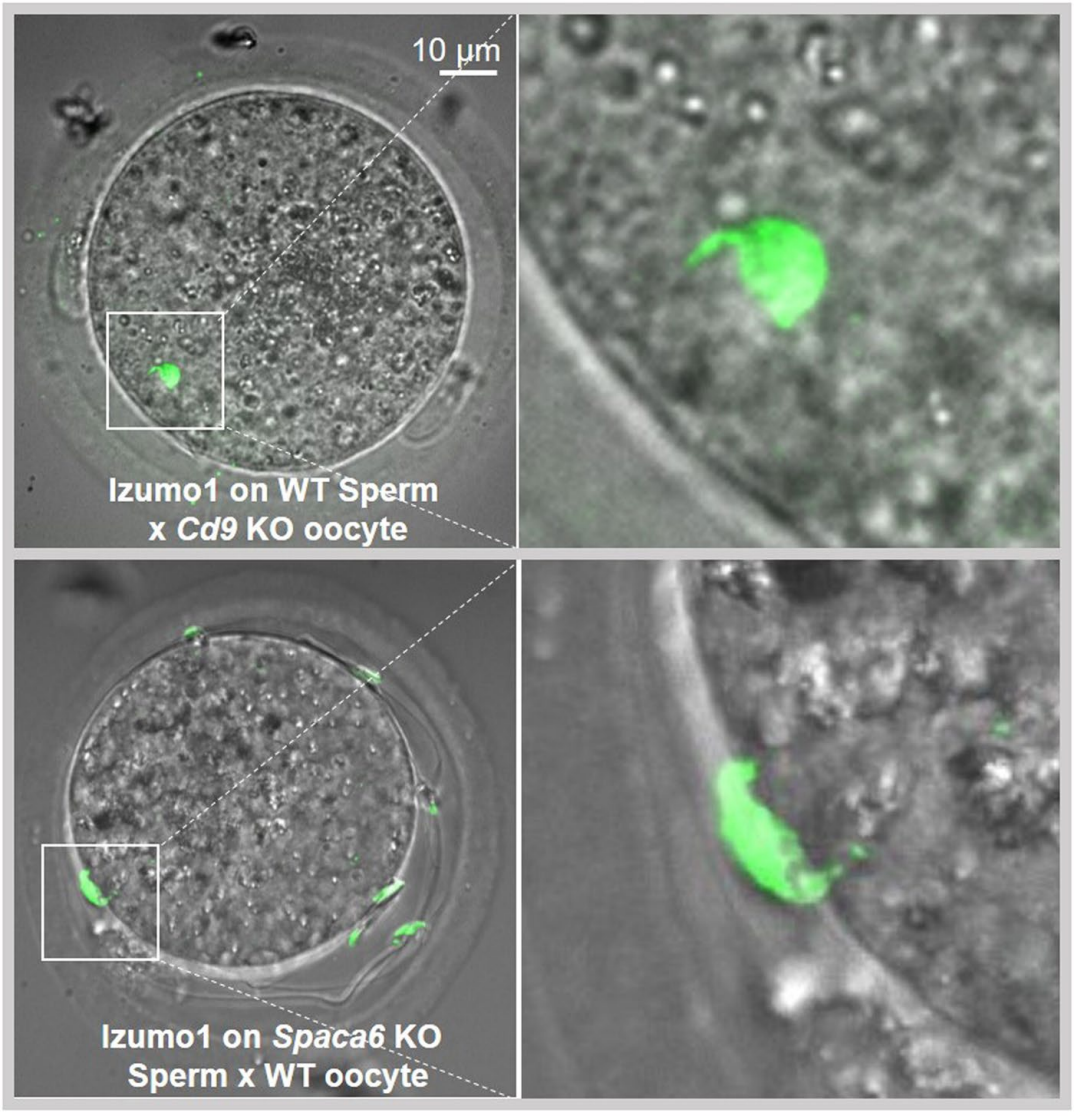
Normal sperm IZUMO1 localization after physiological acrosomal reaction in *Spaca6* KO context. Immunofluorescence staining of IZUMO1 of sperm obtained from WT and *Spaca6* KO C57BL/6 mice after mating with *Cd9* KO and WT females respectively and recovery of oocytes with physiologically acrosome-reacted sperm accumulated in the perivitelline space. The labeling of IZUMO1 (green) was carried out using a rat monoclonal anti-mouse IZUMO1 antibody, recognized by a secondary goat anti-rat antibody coupled to Alexa Fluor 488. Observation was performed under confocal Spinning Disk microscope (IMAG’IC facility). Zooms of the region with a spermatozoon indicate the IZUMO1 distribution. In the perivitelline space, IZUMO1 labeling is present along the entire head, of the WT and *Spaca6* KO sperm heads meaning that even in the absence of SPACA6, the relocation of IZUMO1 after acrosomal reaction occurred normally.

As the dynamic movement of IZUMO1 during acrosome reaction is known to be essential for gamete fusion during mouse fertilization, we explored its redistribution under physiological conditions in the context of *Spaca6* KO. We mated *Spaca6* KO males with WT females and checked the vaginal plugs the day after. We recovered the oocytes containing PVS sperm from the oviduct and immuno-stained them with an anti-IZUMO1 antibody. Controls were obtained by mating WT males with *Cd9* KO females. In the two cases, sperm IZUMO1 was relocated from the acrosomal cap to the whole head indicating that the absence of SPACA6 does not influence the relocation of IZUMO1 following a physiological acrosomal reaction (Fig. 8). Together these results suggested that the role of SPACA6 appears to be independent of the localization and relocation of IZUMO1.

## Discussion

Fertilization is a dynamic process in which the oocyte and the sperm adhere and then fuse to form the embryo. In addition to the three molecular actors described as essential for the gamete adhesion/fusion step during mammalian fertilization, which are the oocyte CD9, organizer of molecular complexes, sperm IZUMO1 and oocyte JUNO, a ligand-receptor pair, a fourth sperm actor has been suggested playing an important role during these steps: SPACA6, sperm acrosome associated 6. A human DNA transcript encoding a putative human homologue of mouse *Spaca6* has been identified on chromosome 19 and human *SPACA6* mRNA was found to be expressed exclusively in testis^18^. *SPACA6* has long been considered as a pseudogene and this is probably the reason why there is no tool to study its function. We therefore tried to produce antibodies directed against the human and mouse proteins. Unfortunately, the one directed against the murine form of the protein proved to be totally non-specific. This is probably due to the polyclonal nature of our antibodies but also to the fact that the protein belongs to a superfamily, that of immunoglobulins, which are known to be involved in cell adhesion. This lack of specificity was raised by the study of mouse *Spaca6* transfected COS-7 cells and confirmed by *Spaca6* deleted sperm analysis. Conversely, COS-7 cells expressing the human form (hSPACA6) made it possible to show that the antibody directed against the human SPACA6 was specific. It is therefore by using this antibody in Western blot analysis that we have shown the presence of the SPACA6 protein on human sperm. The non-detection by immunofluorescence analysis of SPACA6 on fresh and intact (non-permeabilized) sperm indicated that SPACA6 is not localized on the plasma membrane of fresh spermatozoa but is hidden under the plasma membrane and accessible only after the acrosome reaction or permeabilization. As described for IZUMO1^19–21^, we could observe the relocation of SPACA6 during acrosome reaction from the acrosomal cap region to the equatorial segment, where fusion initially takes place. These data are consistent with a role of SPACA6 in the adhesion/fusion steps. We then used functional tests for human zona-free IVF assay in the presence of this antibody and proved its ability to inhibit fertilization. This is the first evidence of a role of SPACA6 in human fertilization. However, this inhibition was not complete, meaning that either the antibody is not completely blocking or that SPACA6 is not essential. Further work with antibodies directed against various regions of SPACA6 is needed to determine the region of SPACA6 involved in fertilization. The ultimate evidence that SPACA6 is essential for human fertilization would be to find a few familial cases or several infertile isolated patients with a mutation in the *SPACA6* gene. In mice, such mutations can be induced, so we have generated two different genetic background KO mouse lines that do not express SPACA6. In the absence of a valid antibody, the lack of *Spaca6* expression has been confirmed at the mRNA level, we also tested several tissues that proved to be negative for the *Spaca6* expression. Lorenzetti *et al*. had already published their work on a *Spaca6* KO mouse, but this mouse no longer exists and the deletion concerned a chromosomal region that did not contain only the *Spaca6* gene^18^. In addition, compared to the other three proteins described as necessary for fusion adhesion (IZUMO1, CD9, and JUNO), and for which there are still many elements to discover about their mechanisms of action, nothing is known about SPACA6 yet. These two KO lines confirmed the sterility of *Spaca6* KO males *in vivo*. *In vitro*, no fertilization was obtained in cumulus-intact and zona-free IVF assays using sperm from KO homozygous males. *In vivo*, heterozygous males behaved as WT males. In contrast, *in vitro*, if no difference was recorded in cumulus-intact IVF assay on the C57BL6/J background, a significant decrease in FR was obtained when we compared the FR of FVB/N sperm from WT and the heterozygous males. This difference between the two genetic backgrounds persisted in IVF zona-free assay in term of FR but disappeared when the FI was compared, since, in both genetic backgrounds, FI with sperm from heterozygous males was intermediate between the values obtained for WT and KO. This intermediate position for the heterozygotes was also found when comparing percentages of PVS sperm-containing oocytes or the mean number of sperm per oocyte after cumulus-intact IVF. This difference emphasizes, on the one hand, the probable and expected difference induced by the genetic background^22^ and, on the other hand, the greater sensitivity of the zona-free IVF assay to detect small differences, in this case invisible *in vivo*. This would probably mean that if we increase significantly the number of matings or the number of generations with heterozygous mice, we would eventually reveal differences in fertility. This also underlined the optimal nature of *in vivo* fertilization. *In vivo*, this apparent normality of heterozygotes is related to the passage of proteins and mRNAs through the intercellular cytoplasmic bridges of the syncytium formed by male germ cells during spermatogenesis, thus making genetically distinct sperm phenotypically equivalent^23^. However, our data suggested that this equivalence is not perfect, as evidenced by a higher percentage of PVS sperm-containing oocytes when they came from a heterozygous male compared to those from a WT male, indicating that the sperm that crossed the zona pellucida are not all able to fuse with the oocyte, certainly because of an unsatisfactory SPACA6 expression level. This reflects the existence of at least partial haploinsufficiency.

There are two important points in common between the three genes described as essential to gamete adhesion/fusion and *Spaca6*: sterility of KO male mice for *Izumo1*^1^ and female KO mice for *Cd9*^3–5^ and *Juno*^2^, and the presence of sperm in the PVS. This PVS sperm accumulation highlights two elements: the first is that all the steps that take place before adhesion/fusion (sperm migration in the female genital tract, capacitation, acrosome reaction, crossing of the cumulus cells and the zona pellucida) occur normally and the second is that the adhesion/fusion is the key step in which these four molecular actors are involved. This second point was confirmed by ICSI experiments showing a similar normal development of embryos derived from oocytes microinjected with sperm from either *Spaca6* KO or WT males.

Under physiological conditions, the fertilization of an oocyte by a sperm triggers the cortical reaction. This phenomenon corresponds to the exocytosis of cortical granules, present in the cortex of oocytes, into the PVS. Cortical granules contain, among others, proteases like Ovastacin, which cleaves the ZP2 proteins of the zona pellucida, thus causing the postfertilization block to sperm binding that ensures monospermic fertilization and successful development^24^. When fusion does not take place, as it is the case for the invalidation of four above-mentioned genes, the cortical reaction is not triggered, the zona pellucida is not modified and therefore it is penetrable by other sperm that accumulate in the PVS.

The very close phenotype of *Izumo1* and *Spaca6* KOs, their belonging to the same superfamily of immunoglobulins and the fact that the structural features of IZUMOl are conserved in sperm protein SPACA6^25^ suggest that these two proteins could play similar roles. Experiments showing interactions of IZUMO1 with JUNO on the egg membrane or with cells transfected with JUNO demonstrated the role of IZUMO1 in adhesion^2,16,26,27^. However, SPACA6 does not appear, from the first published data, to induce adhesion. Indeed, SPACA6-expressing COS-7 cells did not adhere to the egg surface^12^. This suggests that these two proteins may play different roles. An argument in favor of this thesis is the non-compensation found in the absence of one or the other of these two genes indicating that no redundancy exists between these two proteins. This would lead to a scenario in which these two proteins would interact, directly or indirectly, within the same protein complex, for example by allowing the recruitment of a third molecular player essential for the actual fusion step. In addition, adhesion persists, whether in the absence of IZUMO1^1^ or SPACA6 (Video S3) while SPACA6 alone does not seem to induce adhesion^12^. This indicates that there are likely other proteins involved in the adhesion step that are either essential, and still to be discovered, or participating, such as ADAMs, integrins and others. The lack of fusion despite persistence of adhesion may be due to a quantitative defect, as it has been shown in the absence of CD9 causing a lower intensity in the adhesion force due to the clustering failure of several molecules^9^, and/or a qualitative defect, because of the lack of an essential molecular actor whose recruitment requires the concomitant presence of IZUMO1 and SPACA6.

Moreover, it has been shown that in the absence of the TSSK6 kinase, the relocation of IZUMO1 during the acrosomal reaction, from the acrosomal cap region to the equatorial segment, is not done and sperm are then unable to fertilize^28^. To detect a possible influence of SPACA6 on the localization and relocation of IZUMO1, we analyzed its testicular expression by immunofluorescence in the presence or the absence of SPACA6, and we observed no difference between these two cases. Under the physiological conditions of the acrosomal reaction, ie after crossing cumulus cells and zona pellucida, the relocation of IZUMO1 on *Spaca6* deleted sperm in the PVS of WT oocytes was normal and identical to that carried out by WT sperm retrieved in PVS of *Cd9* deleted oocytes.

If the essential role of SPACA6 in fertilization is now definitively demonstrated in mice and strongly suggested in humans, new tools such as monoclonal antibodies, nanobodies, purified protein, *Spaca6* tagged expressing transgenic mouse… are needed to elucidate its molecular mechanisms of action, to identify its sperm and/or oocyte partners and to integrate it into the adhesion/fusion model, certainly more complex than that previously imagined with a well-documented ligand-receptor pair, such as IZUMO1-JUNO.

**Figure 9:**
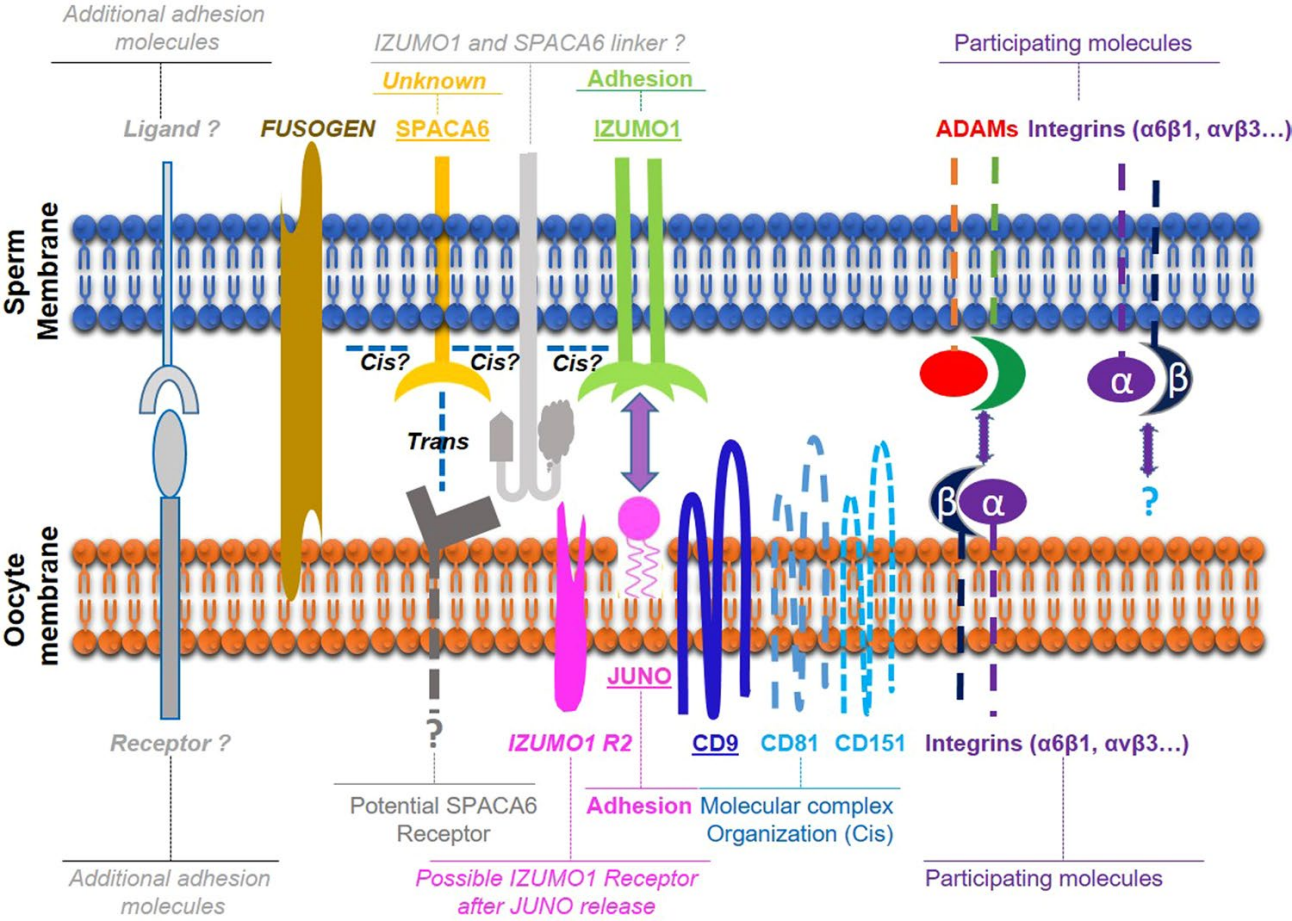
Hypothetical model of mammalian gamete adhesion/fusion. The illustration represents the adhesion/fusion proteins involved in fertilization in mice and humans. The essential ones: SPACA6 and IZUMO1 on the sperm membrane, JUNO receptor of IZUMO1, and CD9 on the oocyte membrane. A non-exhaustive list of some molecules participating but not essential to gamete interaction: ADAMs and integrins on sperm, integrins and tetraspanins on the oocyte. Those that remain to be discovered: the fusogen actor and certainly other ligands and receptors on both sides. We propose a model in which a direct or indirect interaction between SPACA6 and IZUMO1 within a molecular complex would be required to recruit the fusogenic actor.

We propose a hypothetic model (Fig. 9) in which IZUMO1 and SPACA6 would be part of a molecular complex necessary for gamete fusion and that their concomitant presence would be required for the recruitment of another essential molecular actor, such as a fusogen, for the fusion to take place.

## Methods

### Ethics statement

All animal experiments were performed in accordance with national guidelines for the care and use of laboratory animals. Authorizations were obtained from local (C2EA-34, Comité d’éthique en matière d’expérimentation animale Paris Descartes) and governmental ethical review committees via APAFiS Application (Autorisation de projet utilisant des animaux à des fins scientifiques), Authorization APAFIS #14124-2017072510448522 v26, A. Ziyyat (2018–2023).

### Antibodies

Primary antibodies used were: Mouse anti-IZUMO1 monoclonal antibody, IgG2a kappa, clone n°125, (ABIN2452040, anticorps-enligne), was used at 4 µg/ml in immunofluorescence. Rabbit IgG (Invitrogen, CA) was used at 10 µg/ml in immunofluorescence and functional assay. An anti-GFP antibody (A11122, Thermofischer Scientifc) was used at 2 µg/ml in western blot. An anti-human β-tubulin polyclonal antibody (clone AA2, Millipore) was used at 0,3 µg/ml to serve as a loading control.

Rabbit anti-SPACA6 polyclonal antibodies were custom-designed (Eurogentec, Seraing, Belgium) using mouse and human immunogenic peptides: RIRPAQLTHRGTFS and RIRPVQPKHGGTFS respectively, from amino acid 227. They were used at 10 µg/ml in immunofluorescence and functional assay and at 1 µg/ml in western blot.

Secondary antibodies used were: Donkey anti-rabbit Alexa 594 conjugated (A-21207, LifeTechnologies), diluted at 1/500 in immunofluorescence. Goat anti-rat Alexa 488 conjugated (A-11006, Life Technologies), diluted at 1/400 in immunofluorescence. Goat anti-Rabbit HRP conjugated (HAF017, R&D Systems), diluted at 1/1000 in western blot.

### Protein samples preparation

Capacitated sperm samples were washed once in 1X PBS and centrifuged 7 minutes at 600 g. They were resuspended in lysis buffer (20 mM Tris, 150 mM NaCl, 5 mM EDTA, 2 mM EGTA, 2 µg/mL Apoprotin, 1 mM PMSF, 1 mM sodium orthovanadate, 0,5% NP-40, 1X Protease inhibitor cocktail (Roche)), and kept for 30 minutes at 4 °C under agitation. Samples were then centrifuged for 15 minutes at 14000 g, and supernatants were kept. NuPAGE LDS Sample Buffer (ThermoFischer) was added as well as 5% beta-mercaptoethanol. Samples were heated 5 minutes at 95 °C. For each blot, the equivalent of 1 million spermatozoa was loaded on gel.

HeLa and COS-7 cells plated in 6-well plates were transfected using Lipofectamine 2000 reagent (Invitrogen) and 3 µg of plasmids containing the ORF of either human *SPACA6* or mouse *Spaca6* fused to the *GFP* ORF. Pellets of transfected cells were harvested 48 hours post-transfection and extracted in the same conditions.

### Western blot

Samples were loaded in 10% acrylamide gels (home-made). Gels were run for one hour at 150 V. Proteins were transferred from the gel matrix to a PVDF membrane support with a Biorad liquid blotter system (one hour at 70 V). Membranes were blocked one hour in a PBS 1X-Tween 0.1%-Milk 5% solution and placed overnight at 4 °C with the primary antibodies dilution (in PBS 1X-Tween 0.1%-Milk 5%). Membranes were washed 3 times with PBS 1X-Tween 0.1% solution for 10 minutes. They were then placed for one hour at room temperature with the secondary antibody solution in PBS 1X-Tween 0.1%-Milk 5%. Membranes were washed 3 times with PBS 1X-Tween 0.1% solution for 10 minutes. They were then dried and placed for 5 minutes in EMD Millipore Immobilon™ Western Chemiluminescent HRP Substrate (ECL).

### Immunofluorescence analyses of testes, sperm and oocytes

The testes of WT and *Spaca6* KO mice were dissected and instantaneously fixed with 4% paraformaldehyde for 24 h and then transferred to 70% ethanol for 24 h. Paraffin embedding and 4 μm sections were performed on the Histology facility (HistIM) of the Cochin Institute. Sections were deparaffinized in successive baths of xylene and then ethanol followed by unmasking of the antigenic sites using a citrate buffer for 30 min at 95 °C. In order to avoid nonspecific binding with the antibody, a 1-hour block was performed in a PBS BSA 1% solution containing 10% of goat serum. A one-hour incubation at room temperature (RT) with anti-IZUMO1 at 4 µg/ml was performed followed by a second incubation of one hour at RT with an anti-rat Alexa 488 (at 4 µg/ml; Life Technologies®) and Dapi at 5 µg/ml. Slides were observed under a confocal microscope (Leica Spinning Disk). After washing, slides containing testis sections were mounted using a DAKO mounting medium (Dako Fluorescent Mounting Medium, Dako) and covered with a coverslip for observation under UV light (Nikon Eclipse E600 microscope) or a confocal microscope (Leica Spinning Disk).

Spermatozoa were collected and placed in M16 Medium (Sigma-Aldrich) with 3% BSA, then counted on a Malassez chamber. If necessary, spermatozoa were incubated 90 min at 37 °C under 5% CO_2_ air condition to allow capacitation. To induce acrosome reaction, spermatozoa were incubated in the same conditions for 30 more minutes in M16-BSA 3%-10 µM Calcium Ionophore. They were washed 3 times in PBS 1X-BSA 3% for 5 min at 600 g. They were resuspended, fixed in PFA 2% for 30 min and washed 3 times as previously, then incubated overnight at 4 °C with the primary antibody. After 3 washes they were incubated at room temperature with the secondary antibody for one hour. If necessary, after 3 more washes, they were incubated with FITC conjugated PSA (Sigma-Aldrich) for 15 min. After 3 washes, they were resuspended in PBS 1X-BSA 3%, smeared on glass slides and mounted with Fluoromount DAPI mounting medium.

For IZUMO1 detection in PVS sperm, immunofluorescence staining of IZUMO1 and DAPI labeling of C57BL/6 WT and *Spaca6* KO sperm were performed, after mating with *Cd9* KO and WT females respectively and recovery of oocytes with acrosome reacted sperm blocked in the PVS. The labeling of IZUMO1 was carried out using a rat monoclonal anti-mouse IZUMO1 antibody, recognized by a secondary anti-rat goat antibody coupled to Alexa Fluor 488. The genetic material was labeled with DAPI at 5 μg/mL. Observation was performed using confocal Spinning Disk microscope.

DNA content of human eggs was detected by incubating with Hoechst 33342 for 10 min (Thermo Fisher Scientific Massachusetts, USA) at 5 μg/ml followed by several washes in M16 culture medium. Stained oocytes were mounted on Nunc® Lab-Tek® II chambered cover glass (Sigma-Aldrich, Missouri, USA) for confocal analysis using Spinning Disk Microscope (Leica).

### Human gametes and *in vitro* fertilization

Human sperm and oocytes were donated by patients undergoing an assisted reproductive technology (ART) program for *in vitro* fertilization (IVF) or ICSI in the assisted reproductive laboratory of Cochin’s Hospital (Paris, France) after giving informed consent. Only human oocytes that could not be used for the patient fertility purposes were given for research according to the French bioethics laws. Spermatozoa were collected from excess fresh sperm derived from IVF attempts and were used on the day of collection. The GERMETHEQUE Biobank site of PARIS-COCHIN (BB-0033-00081) provided 38 oocytes and 15 samples of sperm. GERMETHEQUE obtained consent from each patient to use their samples (CPP 2.15.27). The GERMETHEQUE pilotage committee approved the design of this study under the number 20160407-01. The Biobank has a declaration DC-2014-2202 and an authorization AC-2015-2350.

*In vitro*-maturated and unfertilized metaphase II oocytes were used for IVF. Zona pellucida was removed chemically using acidic Tyrode’s solution (pH 2.5; Sigma-Aldrich) and oocytes were washed several times in culture medium and kept at 37 °C and 5% CO_2_ atmosphere for recovery. Zona-free oocytes were inseminated in the presence of anti-SPACA6 antibody (10 µg/ml), Rabbit IgG (10 µg/ml) or in their absence with ∼4000 capacitated motile human sperm, pre-incubated with antibody, rabbit IgG or medium respectively during 30 min, in 20 μl Ferticult medium (FertiPro) overnight. For analysis, oocytes were washed and loaded with the DNA-specific fluorochrome Hoechst 33342 (Sigma-Aldrich) at 5 μg/ml for 10 min. After washing, oocytes were mounted between slide and cover slide in Vectashield® Mounting Medium (Vector Labs, California, USA) for observation under UV light (Nikon Eclipse E600) or deposited in a small drop of medium on a Labteck coverslip (Thermofisher) and covered with mineral oil for analysis by confocal microscopy (Spinning Disk). Fusion was considered to have occurred when sperm nuclei were stained with Hoechst 33342 and decondensed.

### Generation and breeding of transgenic mice

*Spaca6* mutant mice were generated by the “Transgenesis and Homologous Recombination” core facility of the Institut Cochin (PRHTEC, INSERM U1016, Paris. France), using the CRISPR/Cas9 technique. Several sgRNA targeting *Spaca6* have been designed and tested in cell lines to assess their efficiency (J-P Concordet, MNHN, Paris) and the RNA guides targeting *Spaca6* exon 1, *5*′*- GCAAACAGCTGGCAGACACGG*-*3*′, and exon 7, *5*′*-* GTACTTCCGTGATATGCCGG-*3*′, were selected. Mice fertilized eggs obtained from superovulated C57BL/6J and FVB/N females have been injected with both sgRNAs and Cas9 protein preincubated to obtain ribonucleoprotein complexes (10 min at RT). The final injection mix contained 0.6 μM of gRNA and Cas9 protein (1.5 μM) in TE-0.1x buffer (10 mM Tris-HCl, 0.1 mM EDTA). Healthy 2-cell stage embryos were subsequently implanted into the oviducts of pseudopregnant B6CBAF1 females. Subsequent genotyping of CRISPR edited founders was performed by PCR amplification (GoTaq® DNA Polymerase, Promega, Madison, WI, USA) on DNA extracted from tail biopsies (NucleoSpin® Tissue, Macherey-Nagel, Düren, Germany) using the following primers respectively flanking guides’ targets (exon1F 5′-CTCTAGCTGGAGCCTGATGC-3′ and exon1R 5′-CCCACTCTGGACCTGTTTCC-3′, exon7F 5′-AGTGCAAGAAGAAGGGTCGG-3′ and exon7R 5′-TCACTGCCAGAGCTCACAAG-3′). A positive amplification between exon1F and exon7R (~428 bp) could sign a deletion of most of the ORF. PCR-product sequencing (Eurofins Genomics, Les Ulis, France) using the amplification primers (Eurogentec, Liege, Belgium) allowed the identification of the precise event. Mice carrying *Spaca6* mutational events were bred with C57BL6/J or FVB/N mice to ensure germline transmission and to obtain heterozygous and then homozygous mutant mice.

When these mice had reached sexual maturity and to test their fertility, *Spaca6* homozygous, *Spaca6* heterozygous and wild-type littermates, 8 to 14 weeks old, were mated with C57BL/6J or FVB/N female mice of 8 weeks. The numbers of pups and litters were recorded.

### Quantitative RT-PCR

RNAs were extracted from fresh mouse tissues using Trizol (Thermo Fischer). They were treated with DNase I (Promega) before reverse transcription with MMLV reverse transcriptase (Invitrogen) in the presence of random hexamers. Real-time PCR was performed using the LightCycler® 480 Sybr Green I Master 2X(Roche) on a LightCycler 480 apparatus. Primers for amplification of *Spaca6* were 5′-GGGAGCATTGTCCTGTTGTT-3′ and 5′-CTTTGAAGGCAAGCCAAAAG-3′ (exons 1 to 3) while amplification of the *Sdha* gene was used for normalization (5′-TCAGTTCCACCCCACAGGTA-3′ and (5′-CTTCTGTGATGAGGCAGCCA-3′).

### Mouse gamete preparation, *in vitro* fertilization and Intracytoplasmic Sperm Injection

#### Oocyte preparation

C57BL6/J or FVB/N female mice of 6–8 weeks (Janvier Labs, France) were superovulated with 5 IU of pregnant mare serum gonadotropin (PMSG) and 5 IU human chorionic gonadotropin (hCG) (Intervet, France) 48 hours apart. About 14 hours after hCG injection, animals were sacrificed by cervical dislocation. Cumulus oocyte complexes were collected by tearing the ampulla wall of the oviduct, placed in Ferticult medium (FertiPro N.V, Belgium) supplemented with 3% BSA (Sigma–Aldrich), and maintained at 37 °C under 5% CO_2_ in air under mineral oil (FertiPro N.V, Belgium). When experiments were performed with zona-free oocytes, cumulus cells were first removed by a brief exposure to hyaluronidase IV-S (1 mg/ml, Sigma–Aldrich). At this step, the obtained metaphase II zona-intact but denuded oocytes can be used for ICSI. The zona pellucida was then dissolved with acidic Tyrode’s (AT) solution (pH 2.5) (Sigma–Aldrich) under visual monitoring. Zona-free eggs were rapidly washed five times and kept at 37 °C under 5% CO_2_ in air for 2 to 3 hours to recover their fertilization ability.

#### Capacitated sperm preparation

Mouse spermatozoa were obtained from the cauda epididymides of mutant or WT C57BL6/J or FVB/N male mice (8 to 10 weeks old) and capacitated at 37 °C under 5% CO_2_ for 90 minutes in a 500 µl drop of Ferticult medium supplemented with 3% BSA, under mineral oil.

#### *In vitro* fertilization

Cumulus-intact and zona-free eggs were inseminated with capacitated spermatozoa for 3 hours in a 100 µl drop of Ferticult medium, 3% BSA at a final concentration of 10^6^ or 10^5^ per ml, respectively. Then, they were washed and directly mounted in Vectashield/DAPI (Vector laboratories, CA, USA) for observation under UV light (Nikon Eclipse E600 microscope). Only oocytes showing at least one fluorescent decondensed sperm head within their cytoplasm were considered fertilized and according to this, the fertilization rate (FR) was evaluated. To assess the fertilization index (FI) in zona-free assay, the number of decondensed sperm heads per oocyte was recorded.

#### Intracytoplasmic Sperm Injection (ICSI)

Metaphase II oocytes, cumulus-free and zona-intact, were prepared as described above. Mouse sperm, obtained from the cauda epididymis of WT or *Spaca6* KO males (10 to 14 weeks old) and capacitated, were sonicated (4 × 5 seconds). This treatment immobilizes sperm and also results in a substantial percentage of acrosome reacted sperm whose tails are clipped off. The separation of heads and flagella was done by gentle centrifugation (300 × g during 5 min). Better survival rates are usually observed when injecting isolated sperm heads instead of a whole spermatozoon because the volume of medium entering the egg is much smaller. During ICSI, a single sperm head was aspirated into a thin glass microcapillary and injected into the cytoplasm of an oocyte on the stage of an inverted microscope fitted with a micromanipulator setup. The crossing of zona pellucida and plasma membrane was facilitated by an electric Piezo (Eppendorf PiezoXpert). After injection, zygotes were kept in KSOM medium (Sigma) allowing *in vitro* embryo development. After 24 hours of culture, 2-cell stage embryos were transferred into pseudopregnant females. The numbers of pups were recorded three weeks later.

### Statistical analysis

Results are expressed as mean ± SEM of at least three independent experiments. For statistical analysis, one-way ANOVA multiple comparisons tests were performed using GraphPad Prism version 7.00 for Windows, (GraphPad Software, La Jolla California USA). Differences were considered statistically significant when p-value <0.05.

## Supplementary information

Supplementary Information.

Video S1.

Video S2.

Video S3.
