# Supplementary material for: Sperm SPACA6 protein is required for mammalian Sperm-Egg Adhesion/Fusion
Source: Sci Rep. 2020 Mar 24;10:5335. doi: 10.1038/s41598-020-62091-y (PMC7093486; doi:10.1038/s41598-020-62091-y)
Supplement: Supplementary file 1 — Supplementary Information. [file 41598_2020_62091_MOESM1_ESM.docx]

**Supplementary Information**

**Sperm SPACA6 protein is required for mammalian**

**Sperm-Egg Adhesion/ Fusion**

Sandrine Barbaux^1^, Côme Ialy-Radio^1^, Myriam Chalbi^1^, Elisa Dybal^1^, Méline Homps-Legrand^1^, Marcio Do-Cruzeiro^1^, Daniel Vaiman^1^, Jean-Philippe Wolf^1,2^ and Ahmed Ziyyat^1,2,*^

1 Université de Paris, Institut Cochin, INSERM, CNRS, F-75014 PARIS, France

2 Service d’histologie, d’embryologie, Biologie de la Reproduction, AP-HP, Hôpital Cochin,

F-75014 PARIS, France

^*^ Correspondence : [ahmed.ziyyat@parisdescartes.fr](mailto:ahmed.ziyyat@parisdescartes.fr)

**Figure S1**


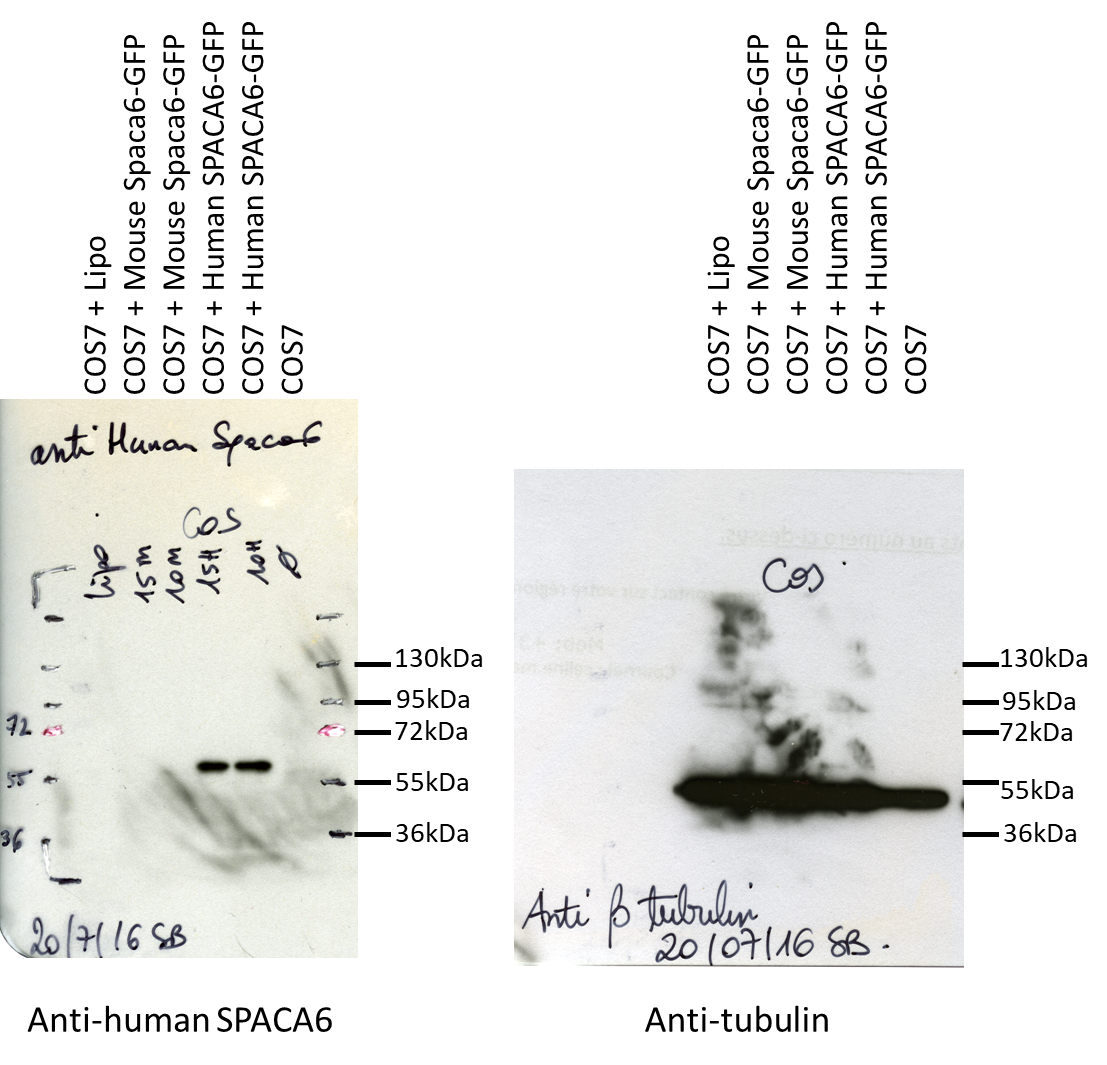


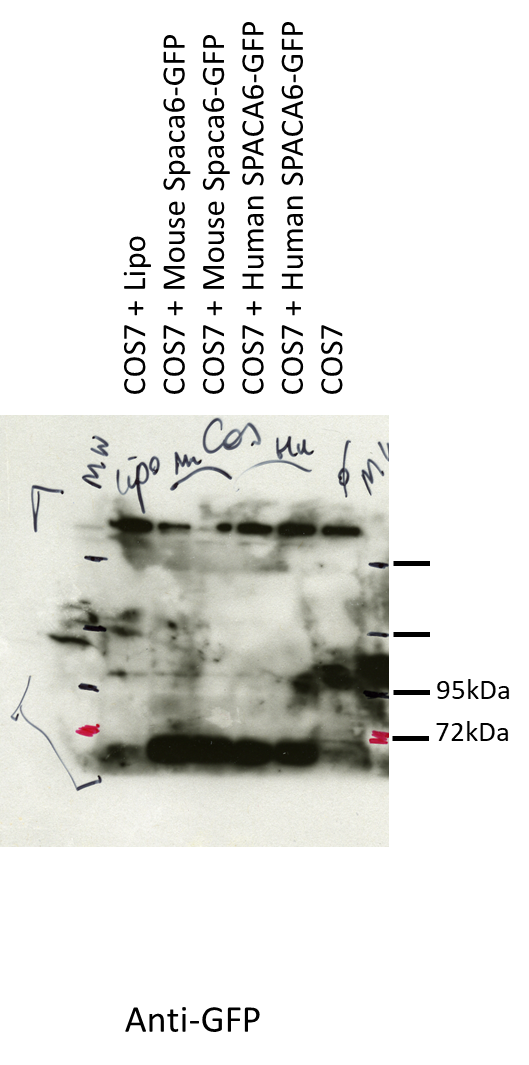


**Figure S1**: **Human sperm SPACA6 expression**

Western blot using extracts of COS-7 cells transfected or not with mouse Spaca6-GFP and human SPACA6-GFP, revealed with rabbit polyclonal anti-human SPACA6, anti-Tubulin and anti-GFP antibodies. A band about 72 kDa (~36 kDa for SPACA6 + ~36 kDa for GFP) was visible only in COS-7 human SPACA6-GFP samples. In contrast, all samples were positive for Tubulin (55 kDa band) and those transfected with GFP were positive for it demonstrating the quality of the deposited proteins and the efficiency of transfection respectively.

**Video S1**

Sperm accumulating and swimming in the perivitelline space of WT oocytes recovered after mating with a C57BL/6 *Spaca6* KO male.

**Video S2**

Sperm accumulating and swimming in the perivitelline space of WT oocytes after cumulus-intact in vitro fertilization with sperm obtained from C57BL/6 *Spaca6* KO male.

**Video S3**

Zona-free IVF assays using sperm derived from C57BL/6 WT and *Spaca6* KO male are shown in parallel. No fertilization was observed with *Spaca6* KO sperm as evidenced by the absence of polar bodies contrary to what seen with WT sperm. However, WT and *Spaca6* KO sperm adhere to the oocytes indicating that the absence of SPACA6 does not completely impede the adhesion step.
